# Training in lung ultrasound for the diagnosis of lower respiratory tract infections in children under five years of age in rural healthcare facilities in Guatemala

**DOI:** 10.7189/jogh.16.04046

**Published:** 2026-02-13

**Authors:** Clara García-Rodríguez, Elena Porras-L, pez, Mercedes Bueno-Campaña, Fleur de Montbel, Isabel Cristina Lobos Medina, Ingris Winter, Ignacio Prieto-Egido

**Affiliations:** 1 Department of Paediatrics and Neonatology, Hospital Universitario del Tajo, Aranjuez, Spain; 2 Department of Paediatrics and Neonatology, Hospital Universitario Fundación Alcorcón, Alcorcón, Spain; 3 Fundación EHAS, Madrid, Spain; 4 Asociación TulaSalud, ONG, Cobán, Guatemala; 5 Signal Theory and Communications, Telematic Systems and Computing Department, Rey Juan Carlos University, Fuenlabrada, Spain; 6 IdiPAZ Research Institute, Translational Research Network for Paediatric Infectious Diseases (RITIP) Madrid, Spain.; 7 Centre for Biomedical Research in Infectious Diseases (CIBERINFEC), Madrid, Spain.

## Abstract

**Background:**

Lower respiratory tract infections (LRTI) in children under five years of age remain a leading cause of mortality, especially in low- and middle-income countries (LMIC). Although many of these deaths are preventable, accurate diagnosis of bacterial pneumonia is essential to ensure appropriate treatment and to reduce unnecessary antibiotic use. Imaging technologies are essential for improving the specificity of diagnoses; however, chest x-rays are often unavailable in rural LMIC settings. In this context, point-of-care lung ultrasound (POCLUS) offers a promising alternative, but the limited training remains a significant barrier to its widespread adoption and long-term sustainability. This study proposes and evaluates a training methodology to address this challenge.

**Methods:**

We conducted a pre-post observational study without a control group across 10 rural health districts in Alta Verapaz, Guatemala. We divided the training programme into four progressive phases: training in technical skills and basic image acquisition (phase 0); assessment of image acquisition skills (phase 1); evaluation of the ability to identify pathological findings (phase 2); and training in a diagnostic algorithm and evaluation in the clinical context (phase 3). In each phase, two specialists independently evaluated images, and a third resolved disagreements. We combined in-person and remote training activities and implemented them between April 2021 and December 2024.

**Results:**

A total of 23 healthcare professionals (six physicians, 15 nurses, and two nursing assistants) participated in the training. Of these, 19 successfully completed phase 1, 18 completed phase 2, and 13 completed phase 3. Eight participants discontinued the programme, primarily due to changes in professional roles or location.

**Conclusions:**

The blended learning model proposed in this study enabled physician and non-physician healthcare providers to use POCLUS to diagnose LRTI in children under five years of age in a rural primary care setting in a low-income country.

Lower respiratory tract infections (LRTI) caused 254 000 deaths among children under five years of age in low-and middle-income countries (LMICs) in 2021 [1,2], with community-acquired pneumonia (CAP) being the most frequent cause of mortality in this age group (16% of the total) [3]. In Guatemala in 2021, LRTIs were the second leading cause of death in children under one year of age (389.39 cases per 100 000 population) and the leading cause of death in children aged 1−4 years (21.77 deaths per 100 000 population) [1].

Although many of these deaths could be avoided through preventive interventions (immunisation, breastfeeding, or nutrition), accurate diagnosis of bacterial pneumonia is essential for adequate treatment, avoiding inappropriate administration of antibiotics. In LMIC such as Guatemala, healthcare in rural areas is provided by physicians and other healthcare professionals (nurses and nursing assistants), and diagnosis is based on the Integrated Management of Childhood Illness (IMCI) strategy recommended by the World Health Organisation (WHO), which prioritises sensitivity [4,5]. Investment in imaging technologies and laboratory tests is thus crucial for enhancing the specificity of diagnoses. Chest radiography, commonly used to confirm the diagnosis of CAP, has a high negative predictive value but low sensitivity and cannot distinguish aetiology. Moreover, in LMICs, the distance to radiology centres can result in delayed diagnosis. In this context, point-of-care lung ultrasound (POCLUS) is presented as a viable alternative. It is a non-ionising technique that is particularly useful and reliable in the paediatric population [6], where the lung mass is small and the chest wall thin, and has high sensitivity and specificity for the diagnosis of CAP [7,8]. From a sustainability perspective, ultrasound offers significant advantages, including low cost, portability, and operational robustness, outperforming other radiological modalities in several aspects [9–11]. It reduces dependence on specialised infrastructure, making it easier to implement in hard-to-reach rural areas [9,12,13]. Although training in the technique and identification of basic pathological images in collaborating patients is simple and can be performed in a short time [14], insufficient training is a known limitation, hindering adoption and sustainability [15–17], and potentially jeopardising diagnostic reliability. Standardised assessments should therefore be incorporated into training programmes, as they provide a framework for evaluating learning outcomes and ensuring the quality of education. Telematic collaborations offer an interesting and effective alternative when quality local training is not available [17]. Finally, although the usefulness of their use in diagnosing LRTI in low-resource settings has been demonstrated, much of the evidence originates from tertiary centres [7,16,18].

We designed a mixed training system, assuming that any health professional with adequate training could use the POCLUS as a diagnostic tool. This system included face-to-face and telematic training, as well as an evaluation of the results. It was aimed at primary care health personnel providing care to paediatric patients in the Alta Verapaz department in Guatemala.

## METHODS

We designed a pre-post observational study without a control group, conducted in the Department of Alta Verapaz, Guatemala, across 10 primary care centres located in 10 rural districts, which we selected in coordination with regional authorities. We did not select participants randomly, as the study involved the healthcare professionals responsible for managing LRTI cases in these districts. These professionals received ongoing training, and we evaluated their learning through several phases.

### Training method design

We organised the training method in four progressive phases, based on individual work (Appendix S1 in the **Online Supplementary Document**).

Phase 0 included basic training (theoretical and practical) on using POCLUS for diagnosing children under five years of age. We began the training with a videoconference session, supported by pre-distributed materials, and followed it with a one-day in-person workshop. Participants then completed three additional days of supervised hands-on practice.

Phase 1 focused on evaluating the adequacy of the technique and the quality of image acquisition in healthy children using the cumulative success rate (CSR). We measured performance at the level of individual cases or patients rather than individual images. For each case, the examiner recorded a total of 10 images per patient. We defined a success threshold as achieving a ‛valid’ rating in at least 80% of the cases.

**Figure 1:**
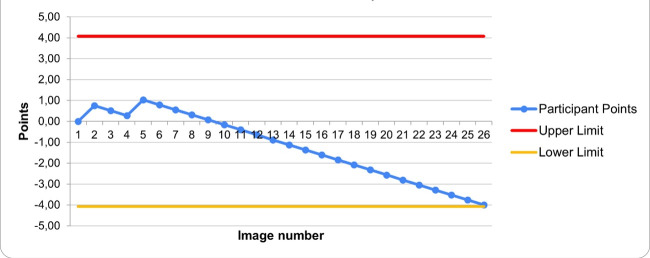
Example of a CUSUM curve generated by one of the study participants.

Phase 2 involved evaluating participants' ability to identify pathological findings using the cumulative sum (CuSum) method [19]. This technique generates a learning curve to determine when participants achieve proficiency and to identify periods of suboptimal performance (**Figure 1**). We established an acceptable (10%) and unacceptable (20%) failure rates, with Type I and Type II error probabilities set at 10%. For participants whose performance crossed the unacceptable failure threshold, we reset the CuSum curve to allow continued monitoring and improvement. We assessed performance based on individual image interpretation and asked participants to submit images with pathological findings.

Phase 3 involved conducting a training in clinical diagnosis using an algorithm designed explicitly for LRTI in children under five years of age, integrating POCLUS into the diagnostic workflow (Appendix S2 in the **Online Supplementary Document**). We based the evaluation on the analysis of ultrasound images and corresponding clinical data to determine whether the diagnosis was correct. We assessed success or failure at the patient level rather than the image level and asked participants to submit the images with pathological findings. The impact of POCLUS on clinical management – particularly regarding treatment appropriateness and reduction in antibiotic use – will be addressed in a subsequent publication.

The training took place between April 2021 and December 2024. We held a three-day face-to-face reinforcement in November 2021 and repeated it one year later, in November 2022. During the first visit, we revisited foundational concepts, addressed questions, and conducted practical workshops. We introduced the phase 3 algorithm, and encouraged participants to ask questions and share feedback, allowing for its adaptation to their specific professional contexts. During the second visit, we aimed to consolidate the knowledge acquired and facilitate the implementation of the diagnostic algorithm defined for phase 3.

In March 2023, we administered a questionnaire to participants who had initiated phase 3 of the training. The survey aimed to gather staff perceptions about evaluating the training methodology, the use of POCLUS, and the barriers that may have affected its implementation. It comprised 20 items rated on a scale from 0 (not at all satisfied) to 5 (very satisfied). We also asked participants to self-assess their competencies in POCLUS and to evaluate its applicability in routine clinical practice (Appendix S3 in the **Online Supplementary Document**).

### Participants

Twenty-three professionals participated in this training, comprising six physicians, 15 nurses, and two nursing assistants. The health authorities were responsible for proposing participants who worked in the public health system and were dedicated to consultations involving paediatric patients with LRTI, rather than other types of consultations or administrative tasks.

### Teaching and evaluation teams

The teaching team comprises three paediatricians trained in POCLUS, from two hospitals in Spain, a radiologist from a hospital in the USA, a coordinator from the local organisation TulaSalud (previously trained in lung ultrasound), and a local radiologist. The three paediatricians and the local radiologist constituted the evaluation team, although the latter only participated in the phase 3 evaluation. The team member from TulaSalud was responsible for monitoring the participants on-site.

At least two paediatricians from the evaluation team interpreted each examination and, in the event of a discrepancy, a third one performed the tie-breaker. In phases 1 and 2, we blinded the evaluators to the clinical presentation. There was no blinding in phase 3, and no independent reference was possible, such as chest radiography.

### Equipment and data collection

Participants used 10 portable ultrasound systems, including a Philips Lumify L12-4 linear transducer and a Samsung A7 tablet running on the Android operating system.

In phases 1 and 2, we collected anonymised ultrasound data in Excel files and shared images through online folders. In phase 3, we stored images on a Picture Archiving and Communication System server implemented with the Orthanc platform [20], both of which are free software tools, and compiled the clinical data in an information system based on DHIS2 [21]. We used a Moodle platform and a videoconferencing system for online training.

### Outcomes variables

We conducted the analysis using aggregated data from the participants, aiming to assess performance across each phase of the experimental protocol. The dependent variables included: the total number of cases or images processed, categorised by phase (cases in phases 1 and 3; images in phase 2); the mean time required to complete each phase; and the percentage of correct responses per phase. This approach enabled a quantitative characterisation of both efficiency and accuracy throughout the procedural stages.

### Ethical approval

We obtained authorisation for the project from the Ministry of Public Health and Social Assistance of Guatemala (official letter number 100-2023. Ref QB/LAJG).

We asked legal guardians of all patients who participated in the various phases to provide verbal consent to obtain the images.

To ensure confidentiality, we anonymised all data and assigned unique identifiers for tracking purposes.

### Statistical analysis

We analysed a total of 349 cases from phase 1, 335 images from phase 2, and 1042 cases from phase 3. For each phase, we calculated the median number of images or cases, the mean time required to complete the phase, and the percentage of correct responses, both in aggregate and stratified by professional category. We summarised Quantitative variables using measures of central tendency (median) and dispersion (interquartile range).

Given the limited sample size, we used nonparametric tests for group comparisons, specifically the Kruskal-Wallis test. After we identified statistically significant differences, we performed *post hoc* pairwise comparisons using the Bonferroni correction. We considered a *P*-value of <0.05 to be statistically significant.

Additionally, to assess improvements within specific groups, we applied the Wilcoxon signed-rank test.

To assess inter-rater reliability among the three evaluators, we calculated the intraclass correlation coefficient (ICC) using a two-way mixed-effects model for numerical assessments in phase 1. For categorical evaluations in phases 2 and 3, we used Fleiss' Kappa measure. We performed all statistical analyses using *R*, version 4.5.1 (R Core Team, Vienna, Austria).

## RESULTS

### Evolution of participants in each phase

Of the 23 professionals who started the process, 13 have passed phase 3 (**Table 1**).

**Table 1:** Breakdown of participants who started, passed or dropped out of each phase

	Start	Abandon	Do not exceed	Exceed
**Phase 1**				
Physician	6		3	3
Nurse	15		1	14
Nursing assistant	2			2
Total	23		4	19
**Phase 2**				
Physician	6	2		4
Nurse	14	2		12
Nursing assistant	2			2
Total	22	4		18
**Phase 3**				
Physician	4	1		3
Nurse	9			9
Nursing assistant	2		1	1
Total	15	1	1	13

At the end of phase 1, three physicians and one nurse did not meet the established criteria. The teaching team agreed that these four participants would move on to phase 2 as they presented more than 70% correct scores. Although their score was not optimal, we considered it acceptable. In addition, at the end of the phase, one participant (physician) withdrew because he had been assigned to another position where he did not provide care to children with LRTI.

We initiated phase 2 with 22 participants, of whom four (two physicians and two nurses) chose not to continue in the programme. Notably, one of them had been allowed to progress from phase 1 despite not meeting the predefined success criteria. In two cases, the CuSum learning curve had to be recalculated due to performance deviations; both participants ultimately met the requirements and continued in the study.

Of the 18 participants who completed phase 2, three withdrew prior to the start of phase 3, and one additional participant discontinued during phase 3. Furthermore, one participant did not meet the success criteria at the end of phase 3, resulting in a final cohort of 13 individuals who completed the full training program. Among them, 10 achieved a diagnostic accuracy rate of 80% or higher. As in phase 1, the success threshold was adjusted to 70% to account for contextual challenges, allowing all 13 participants to be considered as having completed the training.

Phase 1 took place primarily between the last quarter of 2021 and the first quarter of 2022, and phase 2 spanned throughout 2022, extending into part of 2023. Phase 3 started at the end of 2022 peaking in 2024.

### Phase 1

We found the differences between professional categories not statistically significant (*P* > 0.05) (**Table 1**).

During phase 1, we performed a first face-to-face reinforcement, which led to an improvement of 18.8% on average in the percentage of valid results, going from 64.7% (standard deviation (SD) = 25.5) to 83.5% (SD = 20.1), with six more participants exceeding the threshold established in this phase (80%). After training, we found this improvement in results to be statistically significant (*P* = 0.043).

In this phase, the ICC results indicated good to excellent agreement among individual raters (ICC3 = 0.81), and excellent reliability when considering the average of the three raters' scores (ICC3k = 0.95). We found all values statistically significant (*P* < 0.001).

### Phase 2

**Figure 2:**
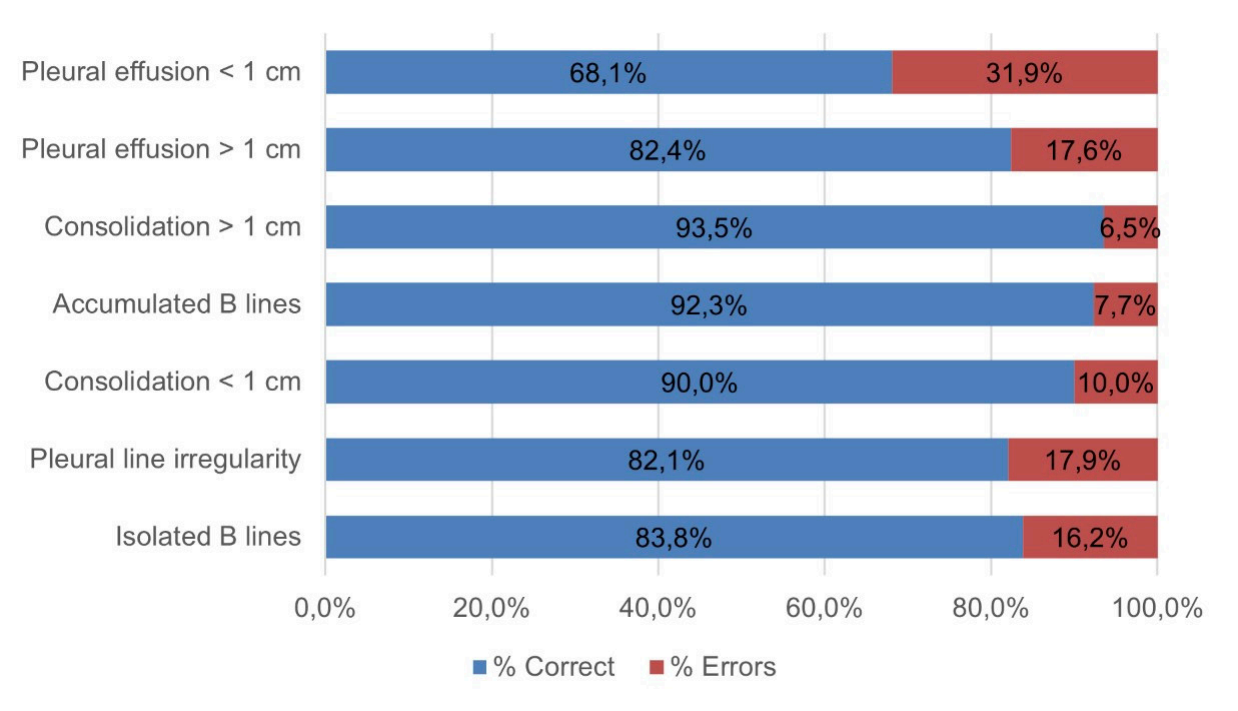
Percentage of hits and misses per finding in phase 2 (over total observations for each lesion).

We observed statistically significant differences between groups for the time variable (*P* = 0.028) (**Table 2**). However, we did not confirm these differences after applying pairwise comparisons, and we found no significant differences for the remaining variables. We also analysed the percentage of hits according to the type of pathological image (**Figure 2**). When testing for differences in accuracy across the various pathologies, the Kruskal-Wallis test yielded a *P*-value of 0.017; however, the *post hoc* analysis did not confirm these differences (*P* > 0.05).

**Table 2:** Global Results of phase 1 and 2*

	Number of studios/images	Time to pass the phase	Percentage of successes
**Phase 1 (n = 349 studios)**			
Physicians (n = 6)	10.50 (14.25)	3.83 (3.79)	76.98 (37.12)
Nurses (n = 15)	14 (7)	4.43 (0.90)	85.71 (26.67)
Nursing assistants (n = 2)	11.5 (min = 11, max = 12)†	4.2 (min = 3.7, max = 4.7)†	91.29 (min = 90.91, max = 91.67)†
Total	12 (7)	4.4 (1.47)	85.71 (26.67)
**Phase 2 (n = 335 images)**			
Physicians (n = 4)	23 (14.25)	8.5 (5.34)	93.65 (9.76)
Nurses (n = 12)	26,5 (5.5)	16.9 (4.51)	90.83 (7.54)
Nursing assistants (n = 2)	29 (min = 29, max = 29)†	13.97 (min = 10.37, max = 17.57)†	89.66 (min = 89.66, max = 89.66)†
Total	27 (5.25)	15.58 (8.17)	90.83 (4.05)

*Values presented as median (interquartile range) unless specified otherwise.

†The interquartile range could not be calculated due to the limited number of cases.

The results revealed moderate agreement between evaluators E1 and E2 (κ = 0.422), between E1 and E3 (κ = 0.469) and between E2 and E3 (κ = 0.638), with *P*-values close to zero, suggesting a reasonable level of consistency in their evaluations.

### Phase 3

**Figure 3:**
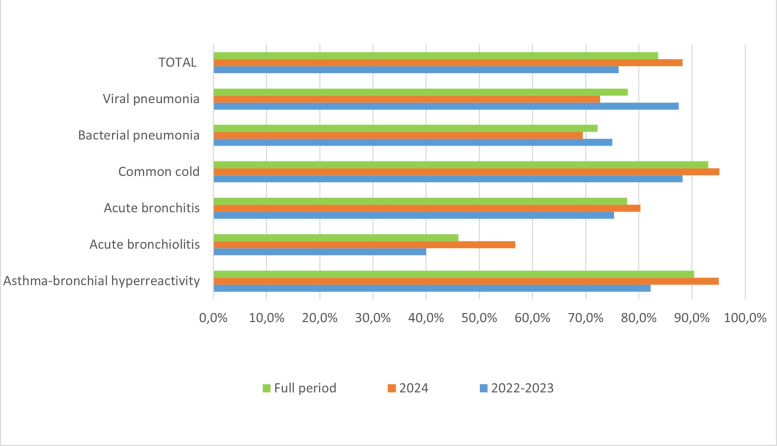
Evolution of correct diagnoses in phase 3 according to pathology.

In phase 3, we analysed a total of 1042 forms corresponding to first consultations. In these, the percentage of correct diagnoses was 83.6%, showing an overall improvement from 76.2% in 2022 − 2023 to 88.2% in 2024 (**Figure 3**). When broken down by pathology, only viral and bacterial pneumonias exhibit a discrete worsening over time. Due to the limited number of cases conducted in 2022, we combined the results from 2022 and 2023, which were not sufficiently representative.

If the overall percentage of correct answers is broken down by profile, physicians had an average of 90.3% correct answers, nursing staff 83.0%, and nursing assistants 68.52% (**Table 3**). The differences between professional categories were not statistically significant (*P* > 0.05).

**Table 3:** Phase 3 global results organised by professional category*

	Number of cases	Time to pass the phase	Percentage of successes
Physicians (n = 4)	80 (218.25)	2.55 (3.28)	91.08 (10.79)
Nurses (n = 9)	29 (79)	7.07 (7.65)	81.82 (14.20)
Nursing assistants (n = 2)	48 (min = 35, max = 61)†	7.5 (min = 1, max = 14)†	68.52 (min = 60, max = 77.05)†
Total (n = 1042)	39 (71)	6.03(8.10)	82.81 (14.62)

*Values presented as median (interquartile range) unless specified otherwise.

†The interquartile range could not be calculated due to the limited number of cases.

The results showed strong agreement among the three international evaluators (κ = 0.818, κ = 0.821, and κ = 0.706), indicating a high level of consistency in their assessments (with *P* close to zero). In contrast, the local evaluator showed poor agreement with the other raters, as indicated by negative or low Kappa values: κ = –0.0135, κ = 0.387, and κ = 0.0541 (*s*>0.05).

### Survey results

Sixteen professionals answered the survey; 87% considered that they had acquired the necessary skills, and 70% rated the evaluation and feedback on the cases provided by the teaching staff as quite satisfactory. Furthermore, 43% identified the length of the procedure as a significant obstacle, and 87.5% recognised lung ultrasounds as a valuable tool (Appendix S4 in the **Online Supplementary Document**).

## DISCUSSION

The proposed mixed teaching method (face-to-face and telematic) has enabled the training of 13 participants (physicians and non-physicians) in a POCLUS programme for the diagnosis of LRTI in patients under five years of age in a rural setting (primary care level) in the Department of Alta Verapaz, Guatemala. Although we have made some adjustments to training limits to compensate for the challenges and difficulties of implementing this technique in a rural setting, the overall analysis suggests that it has been implemented appropriately and has the potential to be effective in this context.

The most common ultrasound training strategy in LMICs has been the occasional visit of experts [11,22–25]. This strategy contrasts with a more structured training model with follow-up. The former can help acquire basic notions, but does not ensure continuity or sufficient practice to face complex cases with confidence. Structured training, on the other hand, improves diagnostic accuracy and promotes the consolidation of skills. In this context, online training emerges as a viable teaching option for LMICs, enabling continuous follow-up and overcoming the need for local experts [15]. Training in paediatric patients presents specific challenges, including difficulties with collaboration and interpretation in complex clinical contexts. Therefore, the methodology should incorporate technical management, image interpretation, and clinical integration [10]. With these principles in mind, we developed a blended, progressive, and structured training programme. This format made it possible to plan face-to-face reinforcement activities in response to detected needs, provide personalised follow-up and conduct continuous evaluation, thereby ensuring higher-quality training.

The CSR is the most widely used method for assessing learning in POCLUS. While it provides insight into general performance trends, it does not directly measure learning progression; rather, it reflects alignment with evaluator interpretations. To address this limitation – particularly in procedures with significant clinical implications – more precise techniques, such as multivariable mixed-effects models [26] or the CuSum method [19], could be employed. Both approaches enable the construction of learning curves that identify when a learner acquires sufficient competence to act autonomously. Despite certain known limitations [27], we selected the CuSum method for phase 2 due to its simplicity and the relatively homogeneous baseline knowledge of the participants.

When considering the raw results from phase 2, we observed the highest diagnostic accuracy to detect accumulated B-lines and consolidations larger than 1 cm – lesions that are technically easier to identify. In contrast, accuracy was lower for pleural effusion and pleural line irregularities, both more challenging to detect but less frequently encountered (**Figure 3**). The CuSum curve employed in this phase incorporates factors such as lesion complexity and frequency, enabling an estimation of the number of ultrasound examinations and the training time required in this context. This approach will facilitate more precise planning of future training sessions, allowing them to be tailored to the specific professional category.

Although the number of cases/images to pass phase 1 and 2 is not high (**Table 2**), the time required to achieve them was considerable (approximately 18 months). This may have been influenced by factors unrelated to the project, which will be discussed later. Moreover, while the length of the process can be seen as an obstacle to the model, it emphasises the importance of more structured long-term training, as opposed to one-off training. Therefore, this is a flexible but demanding system that allows for customised adjustments, but also requires maintaining a minimum level of precision.

After completing phases 1 and 2, it is necessary to evaluate the transfer of skills to clinical practice [26]. In our model, this evaluation corresponds to phase 3. Training in this phase encompasses factors beyond technical expertise, including challenges in obtaining an adequate clinical history, conducting a detailed examination, and interpreting the findings within the clinical context. These additional factors could explain the discrepancies between the high success rate in identifying condensations as isolated pathological lesions in phase 2 (95%) and a lower success rate in diagnosing both viral and bacterial pneumonia in phase 3 (between 70 − 80%). It could also explain the differences in training results between physicians and nursing assistants in phase 3, which were less marked in phases 1 and 2 (**Table 2****,** **Table 3**).

In general, participants reported a positive perception of the training they received. Consistent with previous studies [16], 70% considered the feedback provided to be adequate. In response, we decided to reinforce this aspect, reducing the waiting time for feedback and providing more detailed comments. We identified the main obstacles as daily workload, non-clinical tasks, and the time required to perform the technique and complete the associated forms, as previously noted in other studies [18,28]. We believe that the duration of the procedure can be reduced with practice, and that eliminating digital forms after the training period could further improve efficiency. Finally, as reported in other studies [16,18,28], they recognised POCLUS as a useful and valuable tool, although many admitted not using it regularly.

Nevertheless, nine participants (39%) did not complete the process for various reasons. This dropout rate can be interpreted both as a strength of the method and as a limitation to it: a strength in that it prevents personnel without adequate training from using the technique, and a limitation because the long and demanding training period may make maintaining continuity difficult. However, dropouts are also linked to changes in the destination and role of health personnel, as well as to the lack of institutional support for participants during the project's development. Other added obstacles could be related to the rural working environment, which implies difficulties of travel and communication [28].

The project's strengths lie in its implementation within a rural primary care setting [13] and the learning evaluation methods employed. While the CSR is a commonly used metric, it carries the risk of being influenced by chance, which may lead to an overestimation of competence. In contrast, the CuSum methodology applied in phase 2 offers a more sensitive and dynamic approach, as it incorporates error penalisation and temporal sequencing, thereby providing a more accurate reflection of the complexity inherent in the learning process.

Ultimately, the training method was consistent across all professional categories and yielded very similar results. This supports the applicability of the method in settings where there are few physicians, leaving healthcare in the hands of nurses and nursing assistants. It should be noted that only two nursing assistants participated in the training process, so the results for this professional group should be taken with caution. We incorporated this profile into the project due to the structural shortage of nurses and physicians in the rural districts of Guatemala, where the ratio is 1.3 physicians and 2.3 nurses per 1000 inhabitants, compared to an average of 3.4 physicians and 9.4 nurses in the countries of the Organisation for Economic Cooperation and Development [29]. Future studies should consider incorporating a greater number of nursing assistants.

The absence of a reference standard in phase 3 introduces several methodological limitations. We based the evaluation strategy on consensus between two independent evaluators, with a third reviewer consulted in cases of disagreement. While this approach is recognised as a valid alternative in the absence of a gold standard and is supported by established practices in similar contexts, it may still introduce certain biases.

In our study, we identified two primary potential sources of bias: forced consensus bias, where evaluators are required to assign a diagnosis within predefined categories even when uncertainty exists; and cognitive bias, which may arise from individual evaluators' prior experience, personal beliefs, or professional background. It is important to note that the four evaluators involved represented diverse professional and institutional backgrounds, which may have influenced their diagnostic judgments. However, this diversity also enhances the credibility of the consensus reached, as it reflects a broader range of clinical perspectives.

The high turnover of personnel involved generated operational and structural challenges that could compromise the continuity of the training process and the implementation of the technique [18,28]. Due to the personalised nature of the training, a high level of commitment was required from the professionals, despite the institutional environment not facilitating such dedication or integrating it into a recognised professional development strategy. This lack of alignment between programme requirements and available working conditions may have hindered the consolidation of competencies and the permanence of trained personnel. Due to these difficulties, being pragmatic, we decided to be more lenient in applying the evaluation criteria across the different phases, maintaining an acceptable, though not optimal, training threshold.

Other factors, such as individual variability, statistical parameters (including acceptable and unacceptable error rates), or a small number of cases, may impact the learning curves. There is no universal consensus on the optimal number of cases for a CuSum curve to be representative. In clinical practice and in the surgical learning literature [27], where this methodology has been most studied, it is recommended to analyse more than 50. However, there is no single numerical threshold applicable to all scenarios, and it is recommended to supplement this assessment with other methods. The results obtained in phase 2, with an average of 26 cases, should be interpreted with caution [19] and cannot be considered definitive for high-impact decisions, such as competency certification. In our case, we employed phase 3 to ensure the results were representative, considering both the clinical and educational contexts.

## CONCLUSIONS

The mixed teaching methodology proposed in this study facilitated the training of both physician and non-physician healthcare professionals in the use of POCLUS for diagnosing LRTI in children under five years of age within a rural primary care setting in a low-income country. Although participant numbers declined over time due to high staff turnover, the diagnostic performance of the final cohort of 13 trainees was encouraging.

Replicating this experience in similar contexts may face additional challenges beyond socioeconomic constraints. Institutional factors, such as limited professional recognition, high clinical workload, and competing administrative responsibilities, could hinder the safe and effective implementation of diagnostic innovations like POCLUS, despite their potential to significantly improve patient care.

## Additional material


Online Supplementary Document
