# Supplementary material for: Training in lung ultrasound for the diagnosis of lower respiratory tract infections in children under five years of age in rural healthcare facilities in Guatemala
Source: J Glob Health. 2026 Feb 13;16:04046. doi: 10.7189/jogh.16.04046 (PMC12900549; doi:10.7189/jogh.16.04046)
Supplement: Online Supplementary Document [file jogh-16-04046-s001.pdf]

Supplement to: García-Rodríguez C, Porras-López E, Bueno-Campaña M, de Montbel F, Lobos Medina I, Winter I, Prieto-Egido I. Training in lung ultrasound for the diagnosis of lower respiratory tract infections in children under five years of age in rural healthcare facilities in Guatemala. J Glob Health. 2026;16:04046.

Appendices

Appendix 1. Detailed description of project phases 2
Appendix 2. Description of phase 3 algorithm 4
Appendix 3. Questions of the survey 7
Appendix 4. Results of the satisfaction survey following training in lung ultrasound 9

Appendix 1. Detailed description of project phases

Phase 0: basic training.

Theoretical training by videoconference, with prior delivery of specific documentation prepared for this purpose by the members of the research team located in Spain. Basic classroom training lasting 1 day, as well as 3 days of supervised practice directly at the health centres before the start of the study activities guided by the members of the research team located in Guatemala.

Phase 1

Ensure basic handling of the ultrasound machine and the acquisition of good-quality images. The trainees received remote support from the teachers, who reviewed patient images and provided feedback. Assessment of knowledge and training for progression to phase 2 was by cumulative success rate (CSR).

Inclusion criteria cases phase 1: any patient under 5 years of age who attended consultation for any reason that did not include respiratory pathology.

Items to be assessed in phase 1 evaluation:

1. Initial checklist (must be 100% correctly completed in order to move on to the following item):
    a. Select the "lung" preset in ultrasound.
    b. Correct saving of all images/video clips.
2. Image quality: at least 8/10 valid images must be obtained in each healthy patient studied to be considered successful. The image will be regarded as “valid” if lines A **and** pleural line are seen in at least 60-80% of it.

To consider that the test has been performed successfully in a patient, both items must be fulfilled. If the first point is not 100% fulfilled, the case is considered "NOT VALID".

The total number of images to be recorded is 10 per patient: 5 images with the probe in sagittal/transverse position in the right hemithorax, and 5 images with the probe in sagittal /transverse position in the left hemithorax. The score for each patient goes from 0 to 100%, depending on the percentage of images considered as valid. The final score for each patient will be the average of two evaluators, only if both evaluations exceed 80%. If this is not the case, a third evaluator breaks the tie.

Success or failure will be based on patients (not images)

To pass phase 1, each student must perform ultrasounds on at least 10 patients and achieve a cumulative success rate of 80% or higher. Once 80% of patients studied are labelled "VALID," the student will be considered qualified to move on to the next step.

Phase 2

Identification of pathologic patterns on lung ultrasound images: isolated and/or cumulative B-

lines, pleural line irregularity, condensation less than or greater than 1 cm, simple or complicated pleural effusion:

Inclusion criteria phase 2: patients under 5 years of age with suspected LRTI.

Items to be assessed in phase 2 evaluation:

1. Completion of the checklist (as in phase 1).
2. Description of lesions: isolated B lines, accumulated B lines, pleural line irregularity, condensation smaller than 1 cm, condensation larger than 1 cm, air bronchogram, simple pleural effusion, complicated pleural effusion.

Success or failure will be based on images (not patients). Each of the images with pathology obtained from the same individual will be considered independently for scoring. Success will be considered when point 1 is 100% fulfilled, and at least two evaluators agree upon the image description.

A learning curve (CuSum) will be created with the results of each student considering an acceptable (10%) and unacceptable (20%) failure rate, probability of error type I (10%), and II (10%). For those who crossed the unacceptable failure line, the curve was restarted.

Phase 3

Introduction of clinical ultrasound in the diagnosis of a pathology in a clinical context according to an algorithm designed for this purpose (Appendix 2). In designing the algorithm, the research team considered protocols used in the field—specifically the Integrated Management of Childhood Illness (IMCI) strategy recommended by the World Health Organization (WHO)—along with the professional experience of team members, participant feedback, and various publications supporting the role of point-of-care lung ultrasound (POCLUS) in the diagnosis of pneumonia and bronchiolitis in paediatric populations.

Inclusion criteria phase 3: Patients aged between 2 moths and 5 years old who consulted the primary care centre for a cough lasting less than 7 days.

Information was collected through a digital form that included epidemiological and clinical data, initial diagnosis before ultrasound, location of ultrasound findings, and final diagnosis after the ultrasound. Success or failure will be based on patients (not images). Success will be considered when at least two evaluators agree upon the final diagnosis with the information provided by the participants.

Criteria for passing phase 3: at least 80% of correct diagnoses.

Appendix 2. Description of phase 3 algorithm

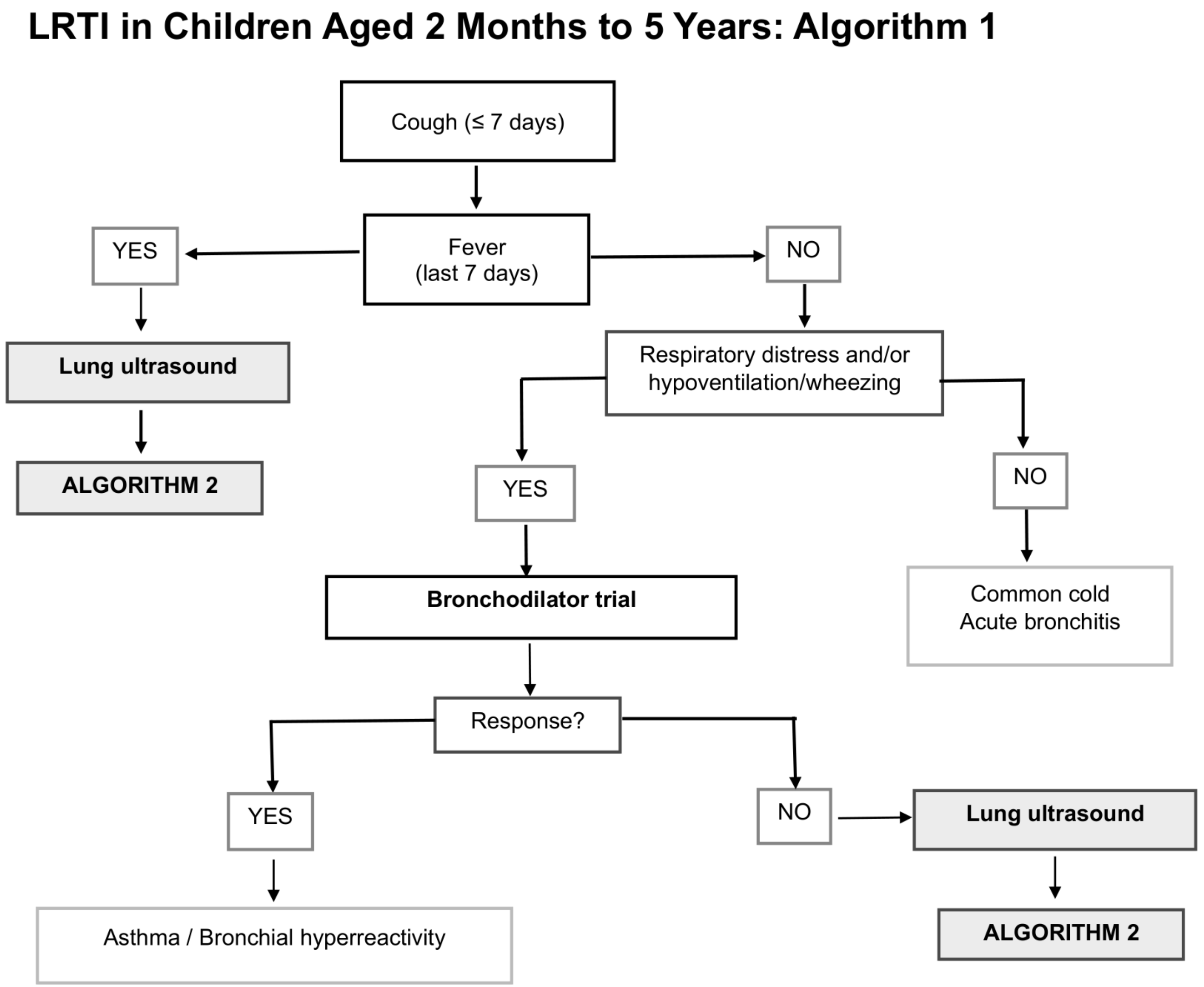

LRTI in Children Aged 2 Months to 5 Years: Algorithm 2

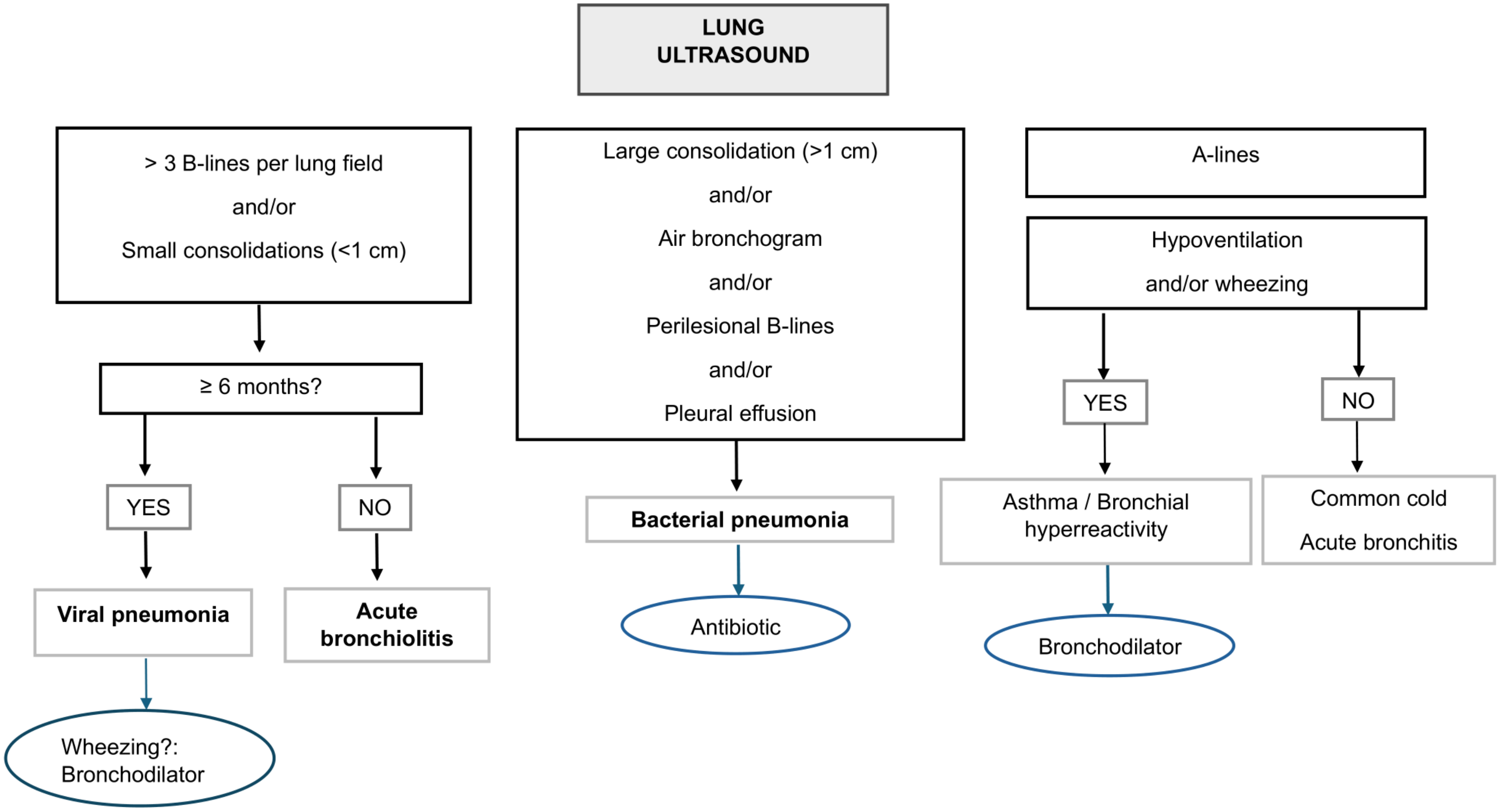

- Berce V, Tomazin T, Gorenjak M, Berce T, Lovrenčič B. (2019) « The Usefulness of Lung Ultrasound for the Aetiological Diagnosis of Community-Acquired Pneumonia in Children. », *Scientific Reports* (2019) 9:17957. https://doi.org/10.1038/s41598-01
- Biagi, C. et al. (2018) « Lung ultrasound for the diagnosis of pneumonia in children with acute bronchiolitis. » *BMC Pulmonary Medicine* (2018) 18, 191, https://doi.org/10.1186/s12890-018-0750-1
- Malla D, Rathi V, Gomber S, Upreti L. (2021) « Can lung ultrasound differentiate between bacterial and viral pneumonia in children? » J Clin Ultrasound. 2021;49:91–100. https://doi.org/10.1002/jcu.22951
- OMS (2022): Pneumonia in Children, World Health Organization; Available from: http://www.who.int/mediacentre/factsheets/fs331/en/
- Pereda, M. A. et al. (2015) « Lung Ultrasound for the diagnosis of pneumonia in children: a meta-analysis. », *Pediatrics* 135, 714–722. https://doi.org/10.1542/peds.2014-2833
- Reissig A, Copetti R, Mathis G, et al. (2012) « Lung ultrasound in the diagnosis and follow-up of community-acquired pneumonia: a prospective, multicentre, diagnostic accuracy study. » *CHEST* 2012; 142(4):965–972
- Testa A, Soldati G, Copetti R, et al. (2012) « Early recognition of the 2009 pandemic influenza A (H1N1) pneumonia by chest ultrasound. », *Critical Care* 2012;16:R30
- Tsung JW, Kessler DO, Shah VP (2012) « Prospective application of clinician performed lung ultrasonography during the 2009 H1N1 influenza A pandemic: distinguishing viral from bacterial pneumonia. », *Critical Ultrasound Journal* 2012;4:16.
- Varshney T, Mok E, Shapiro AJ, Li P, Dubrovsky AS (2016) " Point-of-care lung ultrasound in young children with respiratory tract infections and wheeze". *Emergency Medicine Journal* 2016; 33(9):603.
- Dankoff S, Li P, Shapiro AJ, Varshney T, Dubrovsky AS (2017). "Point of care lung ultrasound of children with acute asthma exacerbations in the pediatric ED". *American Journal of Emergency Medicine*. 2017;35(4):615. doi: 10.1016/j.ajem.2016.12.057. PMID: 28063721.

Appendix 3. Questions of the survey

Concerning the process of training in lung ultrasound.

1.1 Do you consider that you have the skills to perform and interpret a lung ultrasound for children under 5 years old?:

Answer yes or no and justify your answer.

1.2 If your answer was NO, indicate what you would need to consider that you have the mentioned competences:

1.3 Rate from 0 to 5 how confident you feel when performing and interpreting a lung ultrasound in children under 5 years of age, with 0 being not at all confident and 5 being very confident:

1.4 Do you feel you have the skills to perform a correct diagnostic incorporating ultrasound into your clinical assessment?

Answer yes or no and justify your answer.

1.5 If your answer was NO, indicate what you would need to consider that you have the competences mentioned:

1.6. Rate from 0 to 5 how confident you feel when making a diagnosis incorporating ultrasound results into your clinical assessment, with 0 being not at all confident and 5 being very confident.

2. Please rate the training process from 0 to 5, where 0 is not at all satisfactory and 5 is very satisfactory in the following aspects:

- Clarity of the materials
- Clarity of the exhibitors
- Feedback from the team of the project regarding the evaluation of the ultrasound scans
- Accompaniment of the team for the resolution of problems in the filling of the form and
- uploading of the ultrasound scans

3. Do you have suggestions to improve the above or other aspects of the training that you consider? If YES, please list your suggestions.

4. We would like to ask you to send us any comments, appreciation, or proposal for change regarding the algorithm that you are using for the care of children under 5 years of age with ARI, incorporating lung ultrasound scans:

5. How many children under 5 years of age do you see weekly with ARI morbidity?

6. How many lung ultrasound scans do you perform weekly at children under 5 years of age with ARI morbidity?

7. How long does it take you to do a pulmonary ultrasound?

8. Have you had any difficulty in performing these ultrasounds:

If you answered YES, please tell us briefly what challenges you have had?

9. If you answered YES to the previous question, could you tell us what could be improved to overcome the difficulties you have just described?

10. In your experience of care, do you consider that the ultrasound pulmonary has helped you to improve the diagnosis and/or treatment of children under 5 years of age with ARI?

Answer Yes or No and justify your answer.

11. Have you incorporated any changes in your clinical practice thanks to the learning done in the pulmonary ultrasound training?

Answer Yes or No and write down what changes you have incorporated.

12. Have you had any difficulty in applying the algorithm incorporating lung ultrasound in your care of children under 5 years with ARI?

Answer Yes or No. If your answer is Yes, write down what challenges you had.

13. Does the health service have the basic medicines for the treatment of morbidities that are diagnosed?

14. What facilitators do you identify in your health service at the time of performing lung ultrasounds on children with ARI morbidity? List at least two:

15. What obstacles do you identify in your health service when performing lung ultrasounds on children with ARI morbidity? List at least two:

Appendix 4. Results of the satisfaction survey following training in lung ultrasound

1. Clinical Context

A total of 31% (5/16) of respondents reported seeing between 10 and 25 paediatric patients with acute respiratory infections (ARI) per week, while the majority reported seeing fewer than five patients per week. However, this latter group indicated using lung ultrasound more frequently (2–4 scans per week) than those attending to a higher number of paediatric patients.

2. Perceived Competency Acquisition

Self-assessed competency in lung ultrasound included technical skills, recognition of pathological findings, and the ability to integrate these data into diagnostic and therapeutic decision-making. A total of 87% (14/16) of participants reported having acquired the necessary competencies, and 75% (12/16) expressed feeling either fairly confident (4/5) or very confident (5/5) in the clinical application of the tool.

3. Evaluation of Training Materials and Instructor Feedback

A total of 87% of participants rated the training materials as either fairly satisfactory (4/5) or very satisfactory (5/5), with the clinical algorithm receiving particular praise. Instructor feedback was rated as fairly or very satisfactory by 70% (11/16) of respondents. However, three participants noted the need for more in-depth review of incorrect cases, and one participant expressed interest in receiving more in-person support during the training.

4. Technical Aspects

Although the initial uploading of ultrasound cases to the platform was anticipated to be a potential technical challenge, 75% (12/16) of participants reported that this barrier was effectively mitigated through support from the technical assistance team.

5. Barriers to Implementation

A total of 43% (7/16) of respondents identified time constraints as a barrier to implementing lung ultrasound in routine practice. This included the time required to perform the scan (estimated between 15 and 30 minutes), as well as completing the associated documentation, within the context of an already high clinical and administrative workload.

6. Perceived Clinical Utility

A total of 87% (14/16) of respondents acknowledged that lung ultrasound is a valuable tool for improving diagnostic accuracy and therapeutic management of paediatric ARI. Additionally, 43% (7/16) highlighted its role in reducing unnecessary antibiotic use by enabling more accurate identification of pneumonia cases.

7. Complete answers to the survey

Participant ID	Clinical Context					Perceived Competency Acquisition				
	Weekly care for paediatric patients with LRTI	Weekly ultrasounds for paediatric patients with LRTI	Approximate time for ultrasound technique	Difficulty performing ultrasounds	Comments	Competence in performing and interpreting ultrasound images	Confidence in image interpretation (0-5)	Competence for correct diagnosis after ultrasound	Confidence in diagnosis when incorporating ultrasound (0-5)	Comments
1	25	Variable	35	Yes	Improvement with practice	Yes	4	Yes	5	
2	25	0-1	20	Yes		Yes	4	Yes	4	
3	20	1	15	Yes		Yes	3	No	3	Review images
4	18	0-1	15	Yes	Limited availability of ultrasound equipment	Yes	5	Yes	4	
5	10	2	5	No		Yes	5	Yes	5	
6	8	2	30	No		Yes	4	Yes	3	
7	5	3	10	Yes	Poor patient cooperation	Yes	4	Yes	4	
8	4	4	15	No		Yes	3	Yes	3	
9	3	0-1	20	Yes	Poor patient cooperation	Yes	5	Yes	4	
10	2-4	1-3	15	Yes	Fill out the form	Yes	3	Yes	5	
11	2	3	20	No	Poor patient cooperation	Yes	4	Yes	4	
12	2	1	35	No		Yes	4	Yes	4	
13	2	1	25	No		Yes	4	Yes	4	
14	1-2	0-1	30	No		Yes	4	Yes	4	
15	0-1	0-1	25	No	Poor patient cooperation	Yes	4	Yes	4	
16	0-1	0-1	30	Yes	Few patients	No		No	3	

Participant ID	Satisfaction with the material, technical support, and teaching					Usefulness, advantages, and barriers in clinical practice				
	Assessment of the material (0-5)	Teaching assessment (0-5)	Feedback and evaluation of ultrasounds (0-5)	Technical support (0-5)	Comments	Consider ultrasound as an aid in diagnosis and/or treatment.	Incorporating changes into your usual clinical practice after training	What changes has the use of ultrasound brought about?	Difficulty in applying the algorithm	Barriers to implementing ultrasound in your routine practice
1	5	5	5	5	Useful algorithm	Yes	Yes	Better assessment of respiratory work	Sometimes	Little support from centre staff
2	5	5	5	5	Useful algorithm	Yes	Yes	Improvement in auscultation	No	Lack of time Balancing with other tasks
3	4	4	3	3		No	No		Yes	Lack of time
4	1	1	1	1	Useful algorithm	Yes	Yes	Reduce antibiotics	No	Balancing with other tasks
5	2	2	2	2	Useful algorithm	Yes	Yes	Reduce antibiotics	No	Balancing with other tasks
6	5	5	5	5	Useful algorithm	Yes	Yes		No	
7	4	4	3	5		Yes	Yes		Yes	Balancing with other tasks Little support from centre staff
8	4	4	4	5		Yes	Yes		No	
9	5	4	5	5		Yes	Yes		No	Lack of time Balancing with other tasks
10	5	4	4	5	More in-person support	Yes	Yes	Reduce antibiotics	No	Little support from centre staff
11	4	5	4	5	Useful algorithm	Yes	Yes	Reduce antibiotics	No	
12	4	5	5	5		Yes	Yes	Reduce antibiotics	Sometimes	Balancing with other tasks
13	2	2	2	2		Yes	-		No	
14	5	4	4	5	Disagree with some corrections. Useful algorithm	Yes	Yes	Reduce antibiotics. Improvement in bronchodilator indication	No	Lack of time Balancing with other tasks

15	5	5	5	5	Review of cases applying algorithm	Yes	Yes	Reduce antibiotics	Sometimes	Lack of time Balancing with other tasks
16	5	5	4	-	More in-person support	Yes	-		-	
